# Percutaneous transthoracic CT guided biopsies of lung lesions; fine needle aspiration biopsy versus core biopsy

**DOI:** 10.2478/v10019-012-0004-4

**Published:** 2012-01-02

**Authors:** Serif Beslic, Fuad Zukic, Selma Milisic

**Affiliations:** Clinic for Radiology, Clinical Center University of Sarajevo, Bosnia and Herzegovina

**Keywords:** transthoracic biopsy, lung lesions, CT, FNAB, core biopsy

## Abstract

**Background:**

The purpose of this retrospective study was to compare the results and complication rate in CT guided percutaneous trans-thoracic fine needle aspiration biopsies (FNAB) and core biopsies of lung lesions, and to determine the applicability of these needles.

**Patients and methods:**

In 242 patients (166 males; 76 females) with mean age of 58.9 years (13–84 years) CT guided biopsies of lung lesions were performed on dual slice CT equipment. The average diameter of lung lesion was 2.9 cm (1.2–6.3 cm). For FNAB’s 20 – 22 G Chiba needles and for core biopsies 14 G biopsy needles were used. The samples were sent for the histological analysis. The cytological or histological results and the eventual complications were compared.

**Results:**

FNAB’s cytological samples were adequate for definitive diagnosis in 117 patients (79.60 %) and inadequate in 30 patients (20.40 %). Core biopsies samples were adequate in 92 (96.85 %) patients and non- representative (necrotic tissue) in 3 (3.15 %). Pneumothorax as the most frequent complication was detected in 14 (9.7 %) of the patients in the group of FNAB’s and in 30 (31.5 %) of the patients with the core biopsy group.

**Conclusions:**

The results showed that percutaneous transthoracic CT guided biopsies of lung lesions were an effective and safe procedure in the diagnosis of lung lesions. Core biopsy gives a higher percentage of representative samples than FNAB, and is a preferred method regardless of the higher rate of complications.

## Introduction

Percutaneous transthoracic biopsy has become the procedure of choice for diagnosis of pulmonary lesions, which can be primary or secondary malignant tumours.^1^ Although size of lesions, lesions appearance on imaging studies, as well as the patient’s history of smoking, help to assess the likelihood of malignancy, a definitive diagnosis cannot be achieved only on the basis of the image. In the literature some authors prefer thoracotomy or thoracoscopic biopsy of peripheral thoracic lesions, but, like those patients with benign lesions and those with metastatic tumours were exposed to unnecessary surgical procedures.^2^,^3^

Fine-needle-aspiration biopsy (FNAB) with of 20–25 G needle which was first described in 1965 provides a cytological sample of exfoliated cells. Core biopsy with 14–18 G cut needles was described for the first time in the early 1980’s. It has been shown that only 40–50% of small peripheral thoracic lesions are malignant. With percutaneous biopsy a surgery or thoracoscopy could be avoided in 64% of patients.^2^ FNAB is less accurate in the diagnosing benign lung lesions, metastatic lung cancer, mesothelioma and tumours of the anterior mediastinum, and with this method it is more difficult to determine the type of malignoma.^2^–^6^ A negative result of FNAB does not exclude malignancy.

Many authors prefer core biopsy as it provides tissue sample and permits more laboratory testing, such as electron microscopy, immunohistochemistry and analysis of tumour-markers, factors that enhance diagnostic specificity. Large cut needles of 14G have a higher complication rate, while small-sized needles (18G) do not increase the complication rate compared to FNAB.^3^,^7^ It is stated that 18G core biopsy has a higher value than FNAB, for the confirmation of a benign lesion, characterization of malignant cell types, especially in lymphoproliferative diseases (lymphoma), metastatic lung cancer and mesothelioma.^2^,^8^,^9^ Post-biopsy pneumothorax is the most common complication of the percutaneous transthoracic biopsy.^2^,^7^,^10^ It was found that the presence of emphysema and obstructive pulmonary disease, strongly correlate with the occurrence of pneumothorax and the need for drainage. In all cases, the risk of pneumothorax was significantly greater if the lesions were completely surrounded by aerated lung.^5^,^10^,^11^

Haemorrhage is the second most common complication of lung biopsies. It appears as irregular ground glass opacifications, consolidation along or nearby, or in relation with the needle track, immediately after the procedure. Hemorrhagic lesion was considered small if it was ≤ 3 cm, or large if it was ≥ 3 cm in axial diameter.^11^ The small size of the lesion and the long distance to the lesion increase the risk of bleeding. In biopsy of the small lesion, cutting needle often includes a part of aerated lung, having a poorer tampon effect than the solid tissue. When the tumour is deeply located, the needle should pass more aerated lung tissue and pulmonary vessels, increasing the risk of pulmonary hemorrhage.^10^

## Patients and methods

In 242 patients (166 males, 76 females) with mean age of 58.9 years (13 – 84 years) CT guided biopsies of lung lesions were performed on dual slice CT equipment (Emotion Duo, Siemens, Erlangen). Each patient signed an informed consent for a biopsy. All patients had laboratory findings of coagulation factors (prothrombin time and platelets) normal.

A part of the data was collected during the war in Bosnia and Herzegovina and the immediate post-war period. At the beginning of the study, we had only 20–22 G Chiba needles that were used in all patients regardless of the size of the lesion. As soon as we have obtained 14 G cut needles, we decided to use them in all patients and compare the results. We performed FNAB using 20–22 G Chiba needle on 147 patients (group I) and on 95 patients core biopsy with the 14 G cut needle (group II). The sizes of punctured lesions were 1.2 – 6.3 cm (mean 2.9 cm). The puncture entry point was marked on the skin after the sterile preparation, than local anaesthetics of 1% lidocaine was applied.

Three consecutive images were made at the appropriate level, in order to help directing the biopsy needles during the patient’s breath hold. Then the needle is introduced in front of the lesion. We tried to use the shortest intraparenchimal route of the needle, and avoid visible *bulae*. Usually, two passages of the needle were made into different parts of the lesion. Samples obtained by Chiba needle were smeared on a microscope glass slide and sent to the cytological laboratory for Papanicolau or Giemsa staining and analysis. In case of malignant cells the samples were sent for immunohistochemical testing. The samples obtained by core biopsy were fixed in 10% formalin and sent to the patohistological laboratory where they were haematoxylin and eosin, in case of TBC Ziehl-Neelson, PAS, and in case of fungal organisms Grocott’s methenamine silver stained slides were prepared and analyzed.

After the biopsy, a CT scan was performed in order to diagnose potential complications. Patients with no signs of complications were observed for 2 hours, and then discharged. In the case of clinical symptoms (thoracic pain, dyspnoea), or CT evidence of pneumothorax, patients were treated conservatively in minor pneumothorax, or by surgical drainage in severe pneumothorax. The duration of the procedure, pneumothorax, haemoptysis and parenchymal haemorrhage visible on CT scans, were verified. The diagnostic accuracy was evaluated by bronchoscopy and surgical assessment. Non operated patients were observed for at least twelve months with regular CT scans after three, six and twelve months. In case of haematological disease, response to chemotherapy or radiotherapy confirmed the diagnosis. The criteria for benignity in cases where a definitive histological diagnosis could not have been obtained were a three year period of stability, or a regression of the lesion.

## Results

An adequate sample for cytological diagnosis in group I was obtained in 117/147 (79.6%) of patients and in group II histological diagnosis was possible with core biopsy in 92/95 (96.85 %) (Table 1). In both groups more than two-thirds of samples were malignant (in group I 71.51% and in group II 72.66%) and the most common patohistologic diagnosis was adenocarcinoma.

Pneumothorax as the most frequent complication of these procedures occurred in 14/147 (9.7%) of patients in group I and 30/95 (31.5%) of patients in group II.

## Discussion

The FNAB sensitivity in pulmonary lesions is 82 – 99%, specificity 86 – 100%, and the accuracy is 64–97%, depending on whether the cytopathologist was at site, while a definitive diagnosis of benign lesions could be made in only 20–50%.^3^,^7^–^9^ It is generally accepted that FNAB has a diagnostic accuracy over 90% in lung cancer, especially among small-cell and non small-cell cancer.^5^ Accuracy of FNAB in benign lesions is ranged from 12–57%, and usually about 20–30%.^2^ Kocijančič and Kocjančič reported the overall diagnostic accuracy of 93.2% using coaxial 18G Gallini aspiration biopsy needle with cutting tip.^12^ The diagnostic accuracy of FNAB in metastatic lung cancer is only 33%, and in mediastinal tumours, such as lymphoma, thymoma and germ cell tumours is also lower than in case of lung cancer. The number of complications increases with repeated biopsies, although it reduces the rate of false-negative results.^2^ Oikonomou *et al.* reported that core biopsy had a sensitivity 89%, specificity 97%, accuracy 93%, with positive predictive value of 97%, and negative predictive value of 91%. In sub-classification of malignant lymphoma, they found the sensitivity of 85%, specificity 99%, accuracy 92%, positive predictive value 98% and negative predictive value of 87%.^6^

In our study using 14G cut needles for core biopsies, the adequate samples for histological diagnosis were obtained in 98% of biopsies; specificity was 100% and sensitivity 96%. There were 88% true positive core biopsies of malignant lesions, and a specific cell type was identified in 82% of cases. The histological diagnosis was obtained in 66% of biopsies, while 12% were non-diagnostic.^8^

According to some reports, core biopsy is superior to FNAB in diagnosis of benign thoracic lesions, mediastinal tumours, determination of cancer cell-type and predicting cancer-negative findings. As confirmed in our study performing core biopsy and getting adequate samples for patohistological diagnosis it is possible to increase the rate of definitive diagnosis in benign lesions from 52–91%.^2^,^8^,^9^,^13^

Pneumothorax and minor bleeding are the most common complications of transthoracic needle biopsy.^4^ In FNAB the rate of pneumothorax, according to the literature, ranges between 8 and 61%, average 19–44%, while 1.6% – 17% of patients need chest drainage.^5^,^7^,^8^,^10^,^11^,^13^ Performing core biopsies, the incidence of pneumothorax varies from 0% in chest wall and pleural biopsies, to 60% in peripheral intraparenchymal lesions, and close to 100% in small central lesions surrounded by emphysematous *bulae*. The results of our study, performing previously mentioned procedures, FNAB and core biopsy, correspond to the results of other authors.

## Conclusions

The result of our results confirmed percutaneous transthoracic CT guided lung biopsy (both FNAB and core biopsy) as a relatively safe, simple and well tolerated method. Definitive patohistological diagnosis significantly reduces more invasive procedures, cost, hospitalization length and need for surgical diagnostic procedures. According to our experience, core biopsy vs. FNAB provides a higher percentage of patohistological definitive diagnosis and, therefore, is a preferable diagnostic method despite of the slightly higher rate of complications.

## Figures and Tables

**TABLE 1:** Histological or cytological diagnosis of samples taken with fine-needle aspiration biopsy (FNAB) or core biopsy

**Diagnosis**	**Number (%) of patients**	
	**FNAB group**	**core biopsy group**
unrepresentative sample (necrotic tissue)	30 (20.4%)	3 (3.15 %)
malignoma	105 (71.51%)	69 (72.66%)
metastatic nodules	5 (3.43%)	4 (4.21%)
lymphoma	3 (2.04%)	4 (4.21%)
benign lesions	4 (2.72%)	15 (15.77%)
**Total:**	147 (100%)	95 (100%)

**TABLE 2:** Number and type of complications occurred in patients after fine needle aspiration biopsy (FNAB) or core biopsy

**Complication**	**Number (%) of patients**	
	**FNAB group**	**core biopsy group**
pneumothorax	14 (9.7%)	30 (31.5%)
intrapulmonal haemorrhage	13 (9.1%)	14 (14.7%)
